# Acute Small Bowel Evisceration Through Vaginal Cuff Following Intercourse After Total Laparoscopic Hysterectomy: A Rare Surgical Emergency—Case Report

**DOI:** 10.1155/crog/9967414

**Published:** 2025-12-29

**Authors:** Alexander Grainger

**Affiliations:** ^1^ Department of Obstetrics and Gynaecology, Redland Hospital, Brisbane, Queensland, Australia, unicamp.br

**Keywords:** case report, evisceration, post-operative complication, surgical emergency, total laparoscopic hysterectomy, vaginal cuff dehiscence

## Abstract

Vaginal cuff dehiscence (VCD) with bowel evisceration is a rare but serious post‐operative complication following hysterectomy, particularly associated with minimally invasive surgical approaches. It carries significant morbidity and requires prompt diagnosis and surgical intervention. We report the case of a 43‐year‐old woman who presented with post‐coital acute lower abdominal pain and evisceration of bowel through the vagina 3 months after undergoing a total laparoscopic hysterectomy for heavy menstrual bleeding. On examination, large loops of small bowel were noted to be eviscerated through the vaginal canal, with pieces of tissue paper adhered to the eviscerated bowel. Emergent surgical exploration was performed, revealing a dehisced vaginal cuff with underlying viable bowel. The bowel was reduced, the vaginal cuff was repaired transvaginally, and an exploratory laparoscopy was performed with the remaining adherent tissue paper being left on the bowel. The patient recovered without complication and was discharged after a 3‐day hospital stay. VCD with evisceration is a rare complication that requires high clinical suspicion, particularly in posthysterectomy patients presenting with pelvic pain, vaginal bleeding or protrusion. Timely surgical management is essential to avoid bowel ischaemia and other complications. Awareness of risk factors and careful surgical technique during cuff closure at the time of hysterectomy may help prevent this potentially life‐threatening condition. This case also highlights the unique and novel occurrence of tissue paper being left intra‐abdominally at the time of surgery in support of potential conservative management in these instances.

## 1. Introduction

Vaginal cuff dehiscence (VCD) is a rare but often serious post‐operative complication, most commonly occurring following a total hysterectomy procedure. It describes the partial or complete separation of the sutured vaginal cuff which is closed at the time of hysterectomy. It has an estimated incidence of 0.14%–1.4%, with up to 35%–67% of VCD cases involving subsequent evisceration of abdominal or pelvic organs [1–3]. Whilst the estimated incidence of this complication is low, the associated morbidity and mortality are significant and can include potential sequelae of bowel injury, bowel ischaemia, necrosis, infection and sepsis [4].

Early detection and timely surgical intervention are necessary in these cases to replace prolapsed viscera and repair any damage caused in this process. When considering the repair of the vaginal vault, challenges may vary depending on the complexity and timeframe of the dehiscence following hysterectomy. Risk factors include surgical approach (with higher rates noted in minimally invasive hysterectomies), increased intra‐abdominal pressure, poor wound healing, and sexual intercourse [5].

This case report describes a patient who presented with a VCD with evisceration of small bowel loops following a recent hysterectomy procedure. This report is aimed at critically reflecting upon the contributing factors that led to this VCD as well as analyse both clinical and surgical management. Additionally, this report will briefly review the literature surrounding VCD to improve the prevention and treatment of this surgical complication.

## 2. Case Report

A 43‐year‐old gravida 2 para 2 presented to the emergency department after experiencing sudden onset lower abdominal pain and vaginal bleeding following sexual intercourse that evening. The patient reported the sensation of “something protruding” from her vagina and was concerned for pelvic organ prolapse. Her past medical history included well‐controlled hypertension, endometriosis and heavy menstrual bleeding, for which she received a total laparoscopic hysterectomy (TLH) and bilateral salpingectomy 3 months prior. Operation notes revealed an uncomplicated procedure and recovery, with closure of the vaginal cuff using absorbable Stratafix 1‐Polydioxanone (PDS) suture material. This was the first time the patient had resumed sexual intercourse since the operation. Other relevant past surgical history included three previous diagnostic laparoscopies for excision of endometriosis.

**Figure 1:**
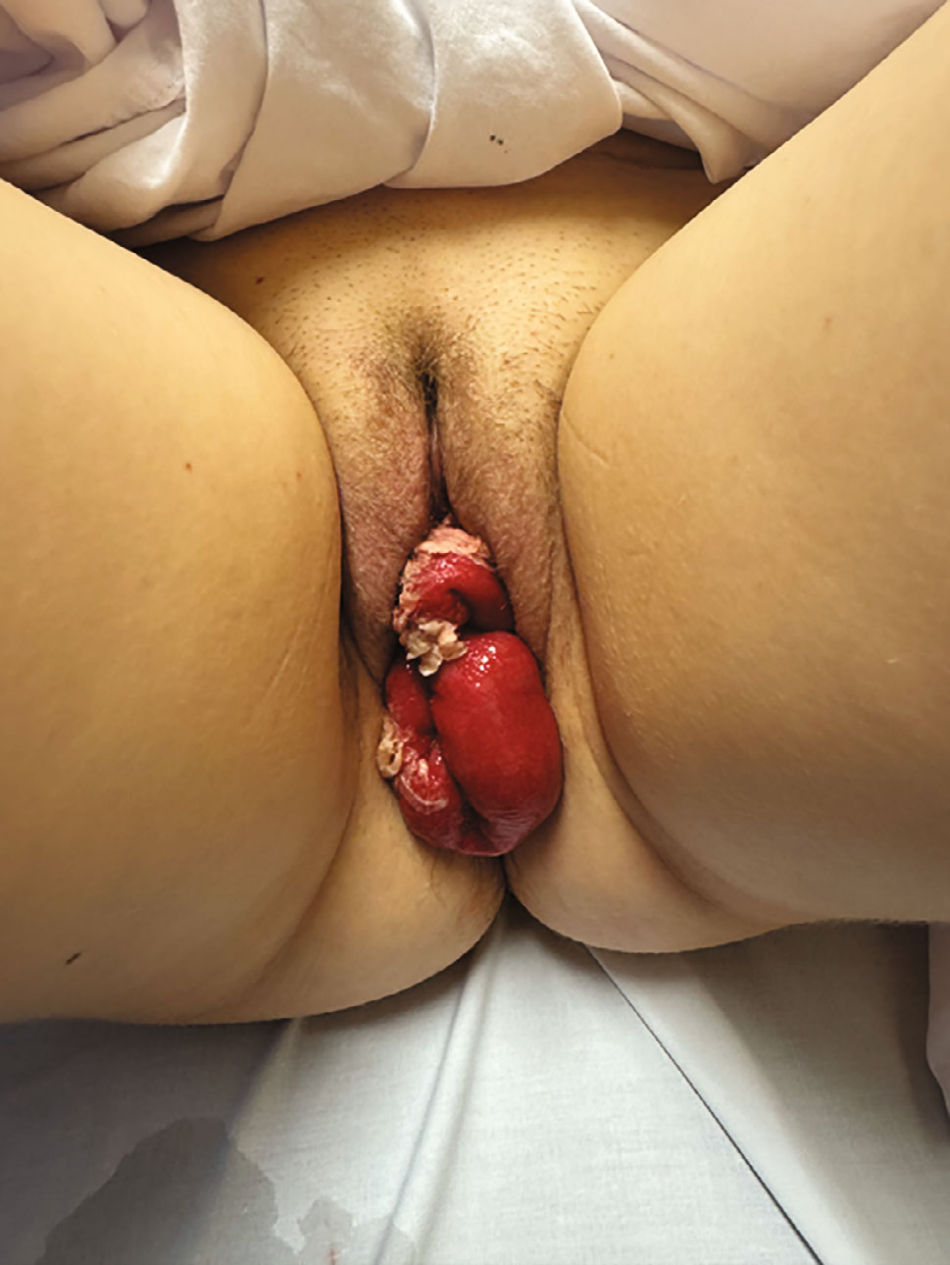
Evisceration of small bowel loops evident through dehiscence of vaginal vault with tissue paper adhered to bowel serosa. Image published with written consent from patient.

Upon initial physical assessment the patient was afebrile and haemodynamically stable, with a normal body mass index (BMI) of 24. Abdominal examination revealed severe tenderness to palpation of the lower abdomen, with extensive guarding but no rebound tenderness. On pelvic examination there was approximately 30 cm of small bowel loops protruding beyond the vaginal introitus as seen in Figure 1. This finding was consistent with a diagnosis of VCD with evisceration. The bowel was pink–red in colour and appeared healthy, without any obvious signs of infection, ischaemia or strangulation. Of note, there was tissue paper densely adhered to the serosa of the small bowel from the patient′s attempt to self‐reduce the evisceration.

Initial serum investigations were collected and did not display any evidence of infection or haemorrhage, except for a mildly raised lactate of 2.4 mmol/L, which, in the context of the patient′s presentation, could be concerning for underlying bowel ischaemia. Reduction of prolapsed small bowel was not attempted in the emergency department and instead the eviscerated bowel was wrapped in isotonic saline‐soaked gauze.

After being reviewed by both general surgery and anaesthetics in the emergency department, the patient was consented for an examination under general anaesthetic, reduction of bowel, repair of vaginal vault and exploratory laparoscopy and taken to the operating theatre as an emergency case. A joint case involving gynaecology and general surgery was undertaken whereby the small bowel was carefully relocated into the abdominal cavity and the vaginal cuff, which was revealed to be completely dehisced, was repaired trans‐vaginally with absorbable 1 Vicryl suture material. Simultaneously, an exploratory laparoscopy was performed, which demonstrated no evidence of bowel or other abdominal viscera injury. Intraoperative cultures were collected, and prophylactic antibiotics were administered and continued for 48 h. The tissue paper that was adhered to the small bowel was attempted to be removed through gentle irrigation and debridement; however, this was subsequently abandoned to prevent any further serosal injury.

Post‐operatively, the patient recovered well, and no further episodes of dehiscence were observed. The patient was discharged home on day three post‐operative with instructions to avoid sexual intercourse and heavy lifting for a further 3 months. At the routine six‐week follow up appointment, the vaginal cuff was healed well on speculum examination and the patient was clinically well. Radiological surveillance with ultrasound revealed no evidence of remnant tissue paper, bowel adhesions or intra‐abdominal collection. The patient was subsequently discharged from the Gynaecology and General Surgery Clinic, respectively.

## 3. Discussion

VCD with bowel evisceration is a rare but serious complication following hysterectomy. The exact incidence of VCD can be difficult to ascertain due to the rare nature of this complication, as well as the limited reported cases worldwide [4]. A brief review of the literature was also performed to provide an up‐to‐date evaluation of potential risk factors and management strategies to improve clinical outcomes of this potentially devastating post‐operative complication.

### 3.1. Hysterectomy Type

TLH has traditionally been thought to be associated with higher incidences of VCD; however, recent meta‐analyses have revealed that robotic‐assisted hysterectomy (RAH) has a higher rate (1.7%) when compared with TLH (0.7%) [6]. Reasons behind this are still unclear; however, the prevailing theory is that a lack of haptic feedback in RAH creates increased potential for excessive force on suture material or lack of tension, increasing the risk of inadequate vaginal cuff closure [7]. VCD after abdominal and vaginal hysterectomy is estimated to have the lowest incidence at 0.2%–0.3% and 0.14%, respectively [8]. In this case, a laparoscopic approach was used and may have contributed to the inherent increased risk of this post‐operative complication through thermal injury from electrosurgical instruments, suturing technique and delayed tissue healing [1].

### 3.2. Primary Vaginal Cuff Closure

Preventative strategies for VCD include meticulous surgical technique, careful tissue approximation and adequate patient counselling regarding post‐operative activity restrictions. Although there are a multitude of risk factors for VCD, the primary surgical factors attributed include the use of energy devices for colpotomy and inadequate cuff closure. Patient factors include premature resumption of sexual intercourse, smoking, diabetes, increased BMI, immunosuppression, poor wound healing and chronic steroid use [8]. Additionally, it has been reported that up to 70% of cases of VCD can occur spontaneously without a known precipitant [9]. Although this patient had no identifiable risk factors besides resumption of sexual activity, the timing and mechanism of injury suggest inadequate tissue integrity at the cuff site, possibly due to delayed healing after laparoscopic closure.

Further studies have investigated surgical factors such as closure technique and suture material for cuff closure. It was found that barbed suture, such as the Stratafix PDS used in this case, was superior to nonbarbed suture in preventing occurrences of VCD [10]. Additionally, the only other statistically significant surgical technique identified in a recent large meta‐analysis of over 19,000 patients was the implementation of a laparoscopic closure of the vaginal cuff instead of vaginal closure [11].

### 3.3. Timing of VCD

The interval between hysterectomy and VCD is highly variable, ranging from 1 month for minimally invasive methods to 5 and 24 months for total abdominal and vaginal hysterectomies, respectively [1]. In this case, the VCD occurred 3 months post the TLH procedure and was provoked by sexual intercourse, which is a well‐known precipitating factor in cases of VCD [12]. Mechanical stress and pressure during sexual intercourse can exert shearing forces on an incompletely healed vaginal cuff, especially in the presence of suboptimal tissue integration or infection. Multiple case series and reviews have identified vaginal intercourse as a leading trigger of posthysterectomy cuff dehiscence, particularly in the early months after surgery [13, 14]. Interestingly, there is an emerging clinical trial in France currently recruiting participants investigating the impact of advising time between hysterectomy and resumption of sexual relations [15]. This is primarily because there are multiple psychological, social and physiological factors involved in female sexual function and to date there is no optimal consensus delineating time between surgery and resumption of sexual intercourse.

### 3.4. Diagnosis and Management of VCD

When considering evisceration as a sequela of VCD, it is noted to have a significantly increased risk of bowel ischaemia, infection and peritonitis, thus necessitating emergent surgical intervention. Understandably, these cases require both early recognition and prompt surgical treatment.

When considering the diagnosis of VCD, clinical suspicion must always remain high in the post‐operative patient. There are several symptoms that these patients may present with; however, the most common include pelvic or abdominal pain (58%–100%) with or without vaginal bleeding/watery discharge (33%–90%) [4]. VCD may also have an associated evisceration in 70% of cases, with the patient reporting a sense of pressure or a mass in the vagina [3]. The most commonly reported precipitating event was sexual intercourse, followed by defecation or Valsalva, with the remainder occurring spontaneously [9]. In our reported case, the patient presented with both abdominal pain and vaginal bleeding, with a feeling of a mass in the vagina, making VCD with evisceration the primary working diagnosis even before clinical examination. It is imperative that all patients presenting following a hysterectomy are appropriately examined to exclude this serious post‐operative complication.

To date, there remains no agreed upon consensus for the optimal method of surgical repair. VCD case reports and cohort studies have demonstrated that 51% of repairs were completed vaginally, 32% abdominally, 2% laparoscopic and 10% combined (abdominal + vaginal or laparoscopic + vaginal), with a further 5% left to heal by secondary intention [4]. The original preference for vaginal repair may have been influenced by limited access to laparoscopic techniques at the time. However, emerging evidence suggests that totally laparoscopic approaches are also safe and effective, and may become the preferred option in the future [16–18]. Additional factors such as patient stability, surgeon expertise, clinical suspicion of bowel injury and the presence of evisceration should also be considered. A combined laparoscopic and vaginal approach was preferred in this case due to clinician experience and ability to permit cuff repair and intra‐abdominal inspection of bowel viability.

### 3.5. A Unique Occurrence

One novel aspect of this case report is the tissue paper that was densely adherent to bowel at the time of surgery. As this tissue paper was unable to be removed due to concerns of causing serosal injury, it was left in situ at time of intra‐abdominal replacement of bowel. To date there are no case reports in the literature that mention retained intra‐abdominal tissue paper and its potential complications. Comparisons from similar case reports and reviews including the cellulose polymer Surgicel and instances of gossypiboma (retained surgical gauze or towel) suggest that any retained foreign material has the potential to cause serious complications including obstruction, peritonitis, abscess and fistula formation [19–21]. With this in mind, it was recommended that this patient receive ongoing clinical and radiological surveillance to assess for any potential further complications. At approximately 6 weeks post repair the patient received an ultrasound, which did not demonstrate any evidence of remnant tissue paper, adhesions or abdominal collection. These findings suggest the potential dissolving of the remaining tissue paper, supporting conservative management as a surgical consideration in future occurrences.

This case highlights the importance of not only thorough education on the prevention of VCD, but also key risk factors and management strategies of this rare surgical complication. After a brief literature review, it is evident that despite an emerging body of management of VCD, no standardised care pathway exists [22]. Further research should be dedicated towards streamlined care pathways and long‐term follow‐up on those who have been successfully treated for VCD.

## 4. Conclusion

VCD with evisceration is a rare but potentially life‐threatening complication of hysterectomy, most commonly encountered following minimally invasive approaches. This case describes an instance of VCD with evisceration, outlining key elements of risk factors, prompt evaluation and effective surgical management. Improved techniques in cuff closure and clearer guidelines for post‐operative activity may further reduce incidence and events of bowel compromise. Evidence‐based management of this serious post‐operative complication is still emerging due to its rarity and requires more case‐based discussions in order to develop a globally accepted guideline to prevent immediate and long‐term complications.

## Consent

All the patients allowed personal data processing and informed consent was obtained from all individual participants included in the study.

## Conflicts of Interest

The author declares no conflicts of interest.

## Author Contributions

Alexander Grainger was involved in patient care, case report conception, literature review and manuscript production.

## Funding

No funding was received for this manuscript.

## Data Availability

The data that support the findings of this study are available on request from the corresponding author. The data are not publicly available due to privacy or ethical restrictions.
